# CTNNAL1 participates in the regulation of mucus overproduction in HDM‐induced asthma mouse model through the YAP‐ROCK2 pathway

**DOI:** 10.1111/jcmm.17206

**Published:** 2022-01-28

**Authors:** Di Wu, Wang Jiang, Caixia Liu, Lexin Liu, Furong Li, Xiaodi Ma, Lang Pan, Chi Liu, Xiangping Qu, Huijun Liu, Xiaoqun Qin, Yang Xiang

**Affiliations:** ^1^ Department of Physiology School of Basic Medical Science Central South University Changsha China; ^2^ Department of Medical Microbiology and Parasitology School of Basic Medical Sciences Capital Medical University Beijing China; ^3^ School of Integrated Chinese and Western Medicine Hunan University of Chinese Medicine Changsha China

**Keywords:** asthma, CTNNAL1, HDM, MUC5AC, ROCK2, YAP

## Abstract

Our previous study indicated that adhesion molecule catenin alpha‐like 1(CTNNAL1) is downregulated in airway epithelial cells of asthma patients and asthma animal model but little is known about how the CTNNAL1 affects asthma pathogenesis. To reveal the direct relationship between asthma and CTNNAL1, CTNNAL1‐deficient mouse model in bronchopulmonary tissue was constructed by introducing CTNNAL1‐siRNA sequence using adeno‐associated virus (AAV) as vector. The mouse model of asthma was established by stimulation of house dust mite (HDM). After HDM‐challenged, there was marked airway inflammation, especially mucus hypersecretion in the CTNNAL1‐deficient mice. In addition, the CTNNAL1‐deficient mice exhibited an increase of lung IL‐4 and IL‐13 levels, as well as a significant increase of goblet cell hyperplasia and MUC5AC after HDM exposure. The expression of Yes‐associated protein (YAP), protein that interacted with α‐catenin, was downregulated after CTNNAL1 silencing and was upregulated due to its overexpression. In addition, the interaction between CTNNAL1 and YAP was confirmed by CO‐IP. Besides, inhibition of YAP could decrease the secretion of MUC5AC, IL‐4 and IL‐13 in CTNNAL1‐deficient 16HBE14o‐cells. Above results indicated us that CTNNAL1 regulated mucus hypersecretion through YAP pathway. In addition, the expression of ROCK2 increased when CTNNAL1 was silenced and decreased after YAP silencing, and inhibition of YAP decreased the expression of ROCK2 in CTNNAL1‐deficient HBE cells. Inhibition of ROCK2 decreased MUC5AC expression and IL‐13 secretion. In all, our study demonstrates that CTNNAL1 plays an important role in HDM‐induced asthma, mediating mucus secretion through the YAP‐ROCK2 pathway.

## 1 INTRODUCTION

Asthma is an airway chronic inflammatory disease characterized by airflow obstruction, bronchial hyperresponsiveness, mucus hypersecretion and airway inflammation.^1^, ^2^, ^3^ It is reported that the airway epithelium is an essential controller of inflammatory, immune and regenerative responses in asthma.^4^, ^5^ In response to allergen stimulation, the airway epithelium secretes fluids, antimicrobial proteins and mucins, which, together with club cells, represent a significant part of the immunomodulatory barrier of the airway epithelium and help orchestrate innate pulmonary immunity.^6^, ^7^, ^8^ Bronchial mucous glands produce mucous plugs within the airway lumen and ectasia of the gland ducts.^9^, ^10^ Mucous plugs are increased in fatal asthma and may contribute to asphyxia due to an abundance of mucous plugs found during the autopsy.^11^, ^12^

Bronchial epithelial cells are the first barrier against environmental pollutants and allergen stimuli.^13^, ^14^ Epithelium dysfunction is involved in the pathogenesis of lung inflammation disorders, such as asthma.^15^, ^16^ Bronchial epithelial cells of asthma patients often show significant structural damage and loss of functional homeostasis. Our previous study showed that the adhesion molecule catenin alpha‐like‐1 (CTNNAL1) was downregulated in asthma patients and in ovalbumin‐stressed asthma mouse model.^17^ Downregulation of CTNNAL1 expression leads to epithelial dysfunction.^17^, ^18^ Airway resistance was highly correlated with CTNNAL1 expression levels.^18^ In addition, CTNNAL1 promoted melanoma progression, metastasis and chemoresistance.^19^ Furthermore, CTNNAL1 correlated with the invasion of breast cancer, prostate cancer and lung cancer.^20^, ^21^, ^22^

It has been demonstrated that CTNNAL1 was identified as a part of the Rho signalling pathway, serving as a scaffold protein for Lbc,^23^ a member of the dbl family of Rho guanine nucleotide exchange factors (GEFs).^24^ Rho GTPases play an important role during the organization of the actin cytoskeleton and the formation of focal adhesion proteins.^25^ Rho‐associated protein kinase (ROCK) is serine/threonine kinase that is downstream target of the GTPases RhoA.^26^, ^27^ There are two isoforms of ROCK—ROCK1 and ROCK2. The two ROCK homologs share 64% identity in their primary amino acid sequences, with the highest homology (92%) within the kinase domains and the coiled‐coil domains being the most diverse (55%). There are tissue‐specific differences in ROCK1 and ROCK2 expression, as well as differences in the subcellular localization of ROCK1 and ROCK2.^27^ It is reported that the ROCK inhibitor, fasudil, decreased the mucus secretion and MUC5AC expression in HDM‐induced mice.^28^

Yes‐associated protein (YAP), a transcription factor, could regulate cell proliferation and differentiation by inducing many genes activation or repression, such as AP‐1^29^ and NR4A1.^30^ In addition,YAP also mediates the integrity of the actomyosin cytoskeleton and the intracellular mechanotransduction pathway. Moreover, it has been reported that YAP interacted with the ROCK2 promoter region in an actomyosin activity‐dependent manner but not ROCK1.^31^

In this study, we demonstrated that CTNNAL1 deficiency caused severer airway inflammation and mucus hypersecretion in response to HDM exposure. Inhibition of ROCK2 could down‐regulate MUC5AC expression when CTNNAL1 was silenced. CTNNAL1 deficiency could promote mucus hypersecretion through YAP‐ROCK2 signalling pathway.

## 2 MATERIALS AND METHODS

### 2.1 Ethics statement

This study was carried out in strict accordance with the recommendations of the Guide for the Care and Use of Laboratory Animals of the National Institutes of Health. The protocol was approved by the Ethics Committee Institute of Clinical Pharmacology of the Central South University. All surgeries were performed under sodium pentobarbital anaesthesia, and all efforts were made to minimize suffering.

### 2.2 AAV synthesis and transfection

The effective AAV5‐CON305 (sequence:TTCTCCGAACGTGTCACGT) and AAV5‐CTNNAL1‐RNAi (sequence:TCAGACAATAACTAAGACA) were designed and synthesized by Shanghai Genechem (Genechem Inc., Shanghai, China). Mice were anaesthetized and intratracheally injected with CTNNAL1‐RNAi‐AAV (containing 10^10^ vector genome copies per injection) or AAV5‐CON305 as control after a week of adjustable feeding.

### 2.3 Animals and challenge protocols

C57bl/6j mice were obtained from the experimental animal center, Xiangya School of Medicine, Central South University. Female C57BL/6 mice, 8 weeks of age and free of murine‐specific pathogens, were obtained and housed under barrier conditions in air‐filtered, temperature‐controlled units under a 12 h light–dark cycle and with free access to food and water. Following the transfection, HDM‐stressed asthma model was constructed according to the previous publication.^32^ The mice were first sensitized to purified HDM extract (10 μg protein in 50 μl saline; Greer Laboratories, Lenoir, NC) via intranasally (i.n.) on Days 14, 21, 28 and 34. The allergen challenge was done via an atomization treatment of HDM on four consecutive days (Day 35–38), and the mice were sacrificed 24 h after the last challenge (Day 39). The control group received sterile PBS via i.n. route and atomization route on Days 14, 21, 28, 34, 35, 36, 37 and 38.

### 2.4 Trachea sampling and treatment

Mouse tracheae were taken from the AAV5‐CON305 and AAV5‐CTNNAL1‐RNAi mice groups. Mouse tracheae were seeded into six‐well cell culture plates and cultured in serum‐free medium. After 24 h of culture, HDM was applied to the culture system for 48 h.

### 2.5 Cell culture, transfection of siRNA, HDM treatment and inhibitor treatment

The 16HBE14o‐cells, a generous gift of Dr. Dieter C Gruenert, University of California San Francisco, are SV40‐transformed human central airway epithelial cells.^33^ Cells were cultured in a mixture medium of DMEM: F12 (1:1) (Sigma, St. Louis, MO) containing 100 μ/ml penicillin, 100 μ/ml streptomycin, 10% foetal bovine serum (Sigma, St. Louis, MO) and incubated at 37℃ in 5% CO_2_. Human bronchial epithelial cells were seeded into six‐well tissue culture plates at an appropriate density and were cultured in a conditioned medium until sub‐confluence. The cells were then washed twice with PBS, and the media was replaced with DMEM containing 5% FBS. After a 24 h culture, the medium was replaced with DMEM containing 10% FBS. The effective CTNNAL1‐siRNA, YAP‐siRNA, ROCK1‐siRNA and nonsense siRNA were synthesized by Guangzhou Ribobio. (Ribobio Inc., Guangzhou, China). At the same time, HBE cells were transfected by silencing siRNA (50 nM) or nonsense siRNA (50 nM) with LipofectAMINE 3000(Invitrogen, Carlsbad, CA, USA) for 24 h. After the transfection for 24 h, the cell culture system was treated with HDM (100 pg, Sigma) for 24 h. KD025 was purchased from MedChemExpress (MedChem Express NJ, USA). KD025(40 μM) and HDM were coadministered to the cell culture system.

### 2.6 Lung histology and immunohistochemical staining

Paraffin‐embedded lung tissue sections were stained using haematoxylin and eosin (HE) (Sigma) or Masson's trichrome. The inflammation score was measured independently by three pathologists blinded to the experiment. The scores from all three were averaged to give a final score.^34^ Morphological changes in fibrotic lungs were quantified according to the criteria proposed by Ashcroft.^35^ Grading was scored on a scale from 0 to 8, using the average of microscope field scores. Immunohistochemical staining was performed on mouse lung paraffin sections using the following antibodies: anti‐CTNNAL1 (Ab96184, Abcam) and anti‐MUC5AC (MA5‐12178, Invitrogen). The mouse lung paraffin sections were incubated with primary antibody and subsequently reacted with relative secondary antibody. The positive cells were brown, and the positive particles were located on the part of antigen. The nuclei were stained blue with haematoxylin. For microscopy, we employed the use of a Moticam Pro Microscope (BA410E, Xiamen, China). Images were obtained via Motic Images Plus3.0 (×64).

### 2.7 Bronchoalveolar lavage fluid (BALF) collection and cell counting

The lungs were lavaged twice with 1 ml sterile PBS to collect one millilitre of BAL (bronchoalveolar lavage) fluid. The BAL fluid was immediately centrifuged at 1500 *g*. The total cell count was measured, and a cytospin preparation was performed. The cells were stained with a Diff‐Quick reagent (Baxter Dade). A differential count of 300 cells was performed using the standard morphological criteria (accurate quantification of cells recovered by BAL lavage).

### 2.8 Scanning electron microscope

Mouse tracheae were taken from the AAV5‐CON305 and AAV5‐CTNNAL1‐RNAi mice groups. Subsequently, the tissues were fixed in cold (4℃) 3% glutaraldehyde in 0.1 M sodium cacodylate buffer (pH 7.4). After 1 h fixation, the samples were trimmed and then transferred to the fresh fixative at 4℃ for another 3 h. The samples were washed in cold (4℃) dilute buffer (equal volume of 0.1 M sodium cacodylate buffer and distilled water); dehydrated in cold condition (4℃) with graded ethyl alcohol in ascending concentrations, ethyl alcohol and acetone in the ratios 3:1, 1:1 and 1:3 and with anhydrous acetone and dried using Critical Point Dryer (E3000 series; Quorum Technologies, East Sussex, UK) with liquid carbon dioxide as the transitional fluid. The tissues were then attached to stubs, coated with gold using Sputter Coater (SC7620; Quorum Technologies) and were examined with a Scanning Electron Microscope (EVO LS 10; Carl Zeiss, Oberkochen, Germany).

### 2.9 ELISA assay

ELISA Kits (Sigma, St. Louis, MO, USA) were used to determine the levels of IL‐4 and IL‐13 according to the manufacturer's protocols.

### 2.10 Overexpression plasmid transfection

The effective CTNNAL1 plasmid was designed and synthesized by Shanghai Genechem (Genechem Inc., Shanghai, China). Transfections were conducted using Lipofectamine 3000 and p3000 (Invitrogen, Carlsbad, CA, USA) in accordance with the manufacturer's instructions.

### 2.11 Rho activity assay

For the Rho activity assay, cells were lysed in RIPA lysis buffer (Sigma‐Aldrich) supplied with a cocktail of protease inhibitors (Roche, Mannheim, Germany). Lysates (500 Ag) were centrifuged at 13,000 *g* for 10 min. The supernatant was rotated for 30 min with 30 Ag GST‐RBD (GST fusion protein containing the Rho‐binding domain of Rhotekin) bound to glutathione‐sepharose beads. Samples were washed in 50 mmol/L Tris (pH 7.4), 10 mmol/L MgCl2, 150 mmol/L NaCl, 1% Triton X‐100 and protease inhibitors. GST‐RBD pull‐downs and lysates were then immunoblotted with an antibody specific for RhoA (Abcam).

### 2.12 Western blot

Proteins from the lung tissues or HBE cells were extracted and analysed using Western blotting as described in our previous study.^36^ Western blotting analysis was performed as described previously. Frozen lung tissue was homogenized and lysed in RIPA buffer (Sigma‐Aldrich) containing a protease inhibitor (Roche, Mannheim, Germany). 50 μg protein that was isolated from bronchial epithelial cells or lung tissue was separated by 10% SDS‐PAGE and transferred to a polyvinylidene difluoride (PVDF) membrane (Millipore, Billerica, MA, USA). The membranes were incubated with primary antibodies as follows: anti‐CTNNAL1 (Ab96174, Abcam), anti‐RhoA (Ab7027, Abcam), anti‐ROCK1 (Ab45171, Abcam), anti‐ROCK2 (#32984, SAB), anti‐MUC5AC (#2692922, Invitrogen), anti‐YAP (AF6328, Affinity), anti‐GAPDH (Ab8245, Abcam) and subsequently reacted with relative secondary antibodies. Film density was measured using ImageJ densitometry software and normalized against GAPDH.

### 2.13 Immunoprecipitation

Immunoprecipitation was performed according to our previous publications.^37^ Briefly, protein from HBE cells was extracted with RIPA lysis buffer (Sigma‐Aldrich). An appropriate dilution of anti‐CTNNAL1 antibody (Ab96174, Abcam) or anti‐YAP antibody (AF6328, Affinity) was added to the centrifuge tube that conjugates with the suspension of Protein G. Mix the antibody–bead mixture at 4°C for 4 h using tube rotator. Then, 50 μg of cell lysates were added into the mixtures and the lysate‐bead/antibody conjugate mixtures were incubated overnight at 4°C. Mixtures were further resuspended in 5xSDS loading buffer. Samples were boiled for 5 min and analysed by Western blot. Immunocomplexes were stained with anti‐CTNNAL1(ab96174, Abcam) as well as YAP (AF6328, Affinity).

### 2.14 Quantitative RT‐PCR

Total RNA was prepared from whole‐lung tissues of mice or human bronchial epithelial cells from different group collected in Trizol (Invitrogen) according to the manufacturer's protocol. Total RNA (1 μg) was reverse transcribed into cDNA using a First Strand cDNA Synthesis Kit (Takara, Japan) following the manufacturer's instructions. SYBR Green signals were detected using a Bio‐Rad real‐time PCR system (CFX96 Touch™, Bio‐Rad, USA). The relative expression of mRNA was determined by normalizing the expression of each gene to glyceraldehyde‐3‐phosphate dehydrogenase (GAPDH) gene following the 2^−ΔΔ^Ct method. Primer sequences are shown in Tables 1 and 2.

**TABLE 1:** Primer sequence of mouse genes for qPCR

Mouse gene	Primer sequence
CTNNAL1	Forward: 5′‐AGATGAGTGACATGGCGACG‐3′
	Reverse:5′‐CAGCCCGAGCTTTGCTATCT
MUC5AC	Forward:5′‐GTCTCCCTGGATGGATGTT‐3′
	Reverse:5′‐ACTGCTTTTGGCACTTGG‐3′
MUC5B	Forward:5′‐TCCTCATCCACAAGGCA‐3′
	Reverse:5′‐CGGAACACGTCACCATC‐3′
RhoA	Forward:5′‐TGGGAAGCAGGTAGAGTTGG‐3′
	Reverse:5′‐GTCTCGTGTGCTCGTCATTC‐3′
ROCK1	Forward:5′‐GTGGTATTGAAAGCCGCACTG‐3′
	Reverse:5′‐TGCCATCTATTCATTCCAGCCAT‐3′
ROCK2	Forward:5′‐TGCTTGCCCTACCTTCC‐3′
	Reverse:5′‐CACCTTTACTCACCGACCA‐3′
IL‐13	Forward:5′‐GAAGCAGTGGGCTCTGG‐3′
	Reverse:5′‐CGTGGCAGACAGGAGTG‐3′
GAPDH	Forward: 5′‐TTGCAGCTCCTTCGTTGCC‐3′
	Reverse: 5′‐GACCCATTCCCACCATCACA‐3′

**TABLE 2:** Primer sequence of human genes for qPCR

Human gene	Primer sequence
CTNNAL1	Forward:5′‐CTACACCAGCCATGAGCA‐3′
	Reverse:5′‐GCCTGAGTTGACAGTTCCA‐3′
MUC5AC	Forward:5′‐AAAACGGCATCGTGGTCT‐3′
	Reverse:5′‐GGCACTCATCCTTCCTGTC‐3′
RhoA	Forward:5′‐TGGAGCTGGGCTAAGTAAA‐3′
	Reverse:5′‐CTCTGGGAGGGAACCTG‐3′
ROCK1	Forward:5′‐CAAACGATATGGCTGGAAG‐3′
	Reverse:5′‐TGGATTGGATTGCTCCTTA‐3′
ROCK2	Forward:5′‐CTGCCTTACACAAAATGACCT‐3′
	Reverse:5′‐CATCTGCATCCTGACGTTC‐3′
IL‐13	Forward:5′‐TAGCCGACCTCAGCCTT‐3′
	Reverse:5′‐TGCCTGTGTGTGAAGTGG‐3′
YAP	Forward:5′‐CCGTTTCCCAGACTACCTT‐3′
	Reverse:5′‐TTGGCATCAGCTCCTCTC‐3′
GAPDH	Forward:5′‐GACGCTCACCCCAGACA‐3′
	Reverse:5′‐TGACACCCCACAGCAAGA‐3′

### 2.15 Statistical analysis

All experiments were run at a minimum of triplicates, and analysis was performed using the PRISM (GraphPad, La Jolla, CA, USA) statistical software. An unpaired t test was used for comparisons between two groups. Differences among multiple groups were determined by ANOVA, followed by Bonferroni correction for multiple comparison testing. All data are presented as the mean values ± standard deviation (SD). *p* < 0.05 was considered statistically significant.

## 3 RESULTS

### 3.1 CTNNAL1‐deficient mouse model was successfully constructed

**FIGURE 1:**
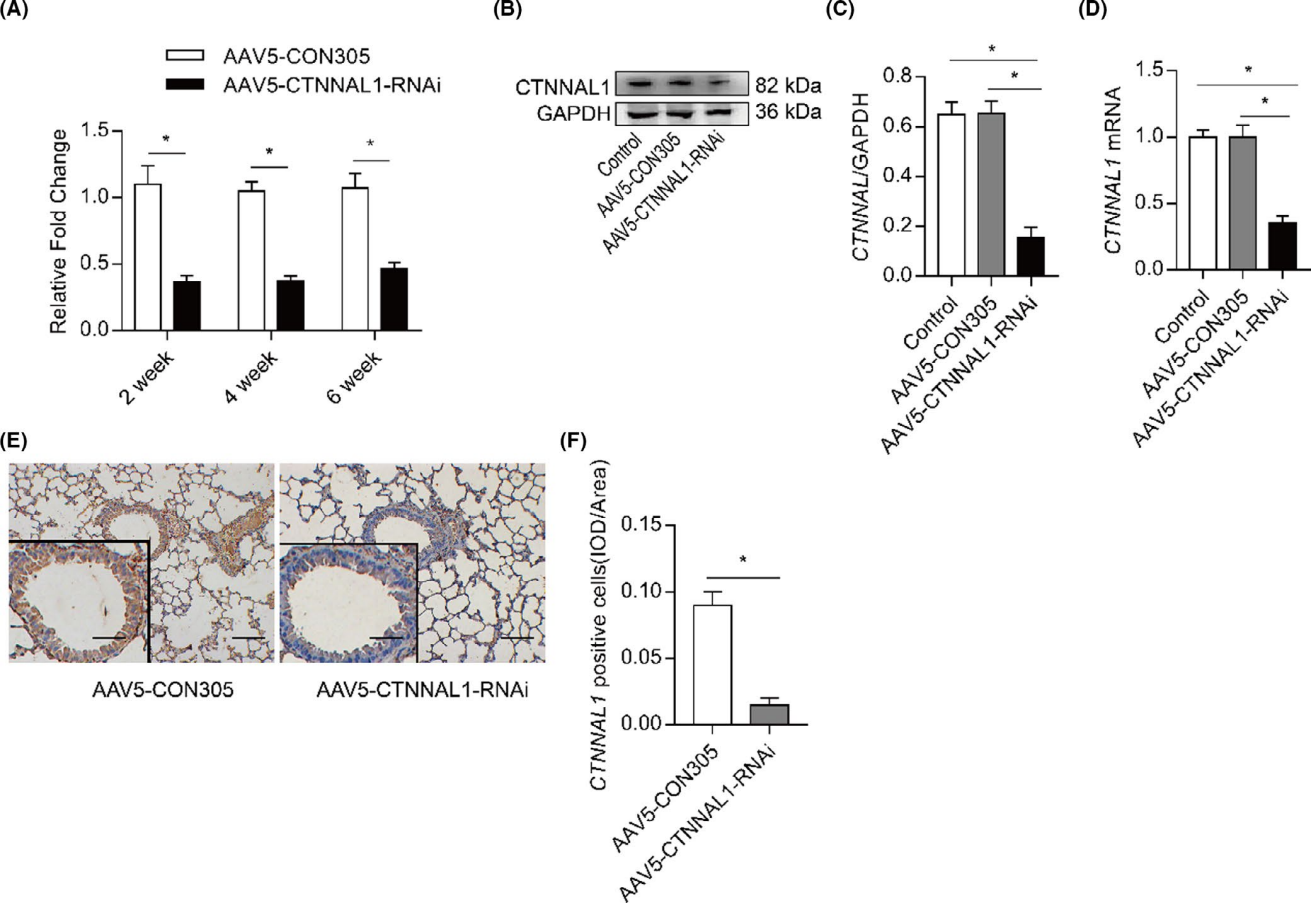
CTNNAL1‐deficient mouse model was successfully constructed. (A) CTNNAL1 mRNA expression in the lungs was detected via RT‐qPCR. Results of AAV5‐CTNNAL1‐RNAi group and AAV5‐CON305 group were quantified relative to mRNA of GAPDH (*n* = 6). (B,C) CTNNAL1 protein expression in the lungs was detected by Western blot. Quantification of Western blotting was normalized to the level of glyceraldehyde 3‐phosphate dehydrogenase (GAPDH) (*n* = 5). (D) Significant inhibition of CTNNAL1 was detected via RT‐qPCR (*n* = 6). (E,F) CTNNAL1 expression in the airway was detected by immunohistochemistry (*n* = 5; ×200, Bars = 100 μm, left panel ×400, Bars = 50 μm). The experiments were performed three times, and the error bars represent means ± SD. *p < 0.05

AAV5‐CTNNAL1‐RNAi was used to silence CTNNAL1 in the lungs and tracheae of the mice. We detected the mRNA expression of CTNNAL1 at 2, 4 and 6 weeks after AAV5‐CTNNAL1‐RNAi injection (Figure 1A). The silencing efficiency of CTNNAL1 at 2 and 4 weeks was greater than that of 6 weeks. In the AAV5‐CTNNAL1‐RNAi mouse group, CTNNAL1 protein expression decreased approximately 65% (Figure 1B,C), and the mRNA expression decreased 60% compared with control and the AAV5‐CON305 mouse group (Figure 1D). We found that the expression of CTNNAL1 was significantly lower than that found in the AAV5‐CON305 group (Figure 1E,F). Transfection of AAV5‐CTNNAL1‐RNAi did not influence the expression of CTNNAL1 in the livers or kidneys (Figure S1B), and did not cause liver or kidney injuries or body weight loss (Figure S1A,C).

### 3.2 CTNNAL1 deficiency promoted airway inflammation in HDM‐treated mice

**FIGURE 2:**
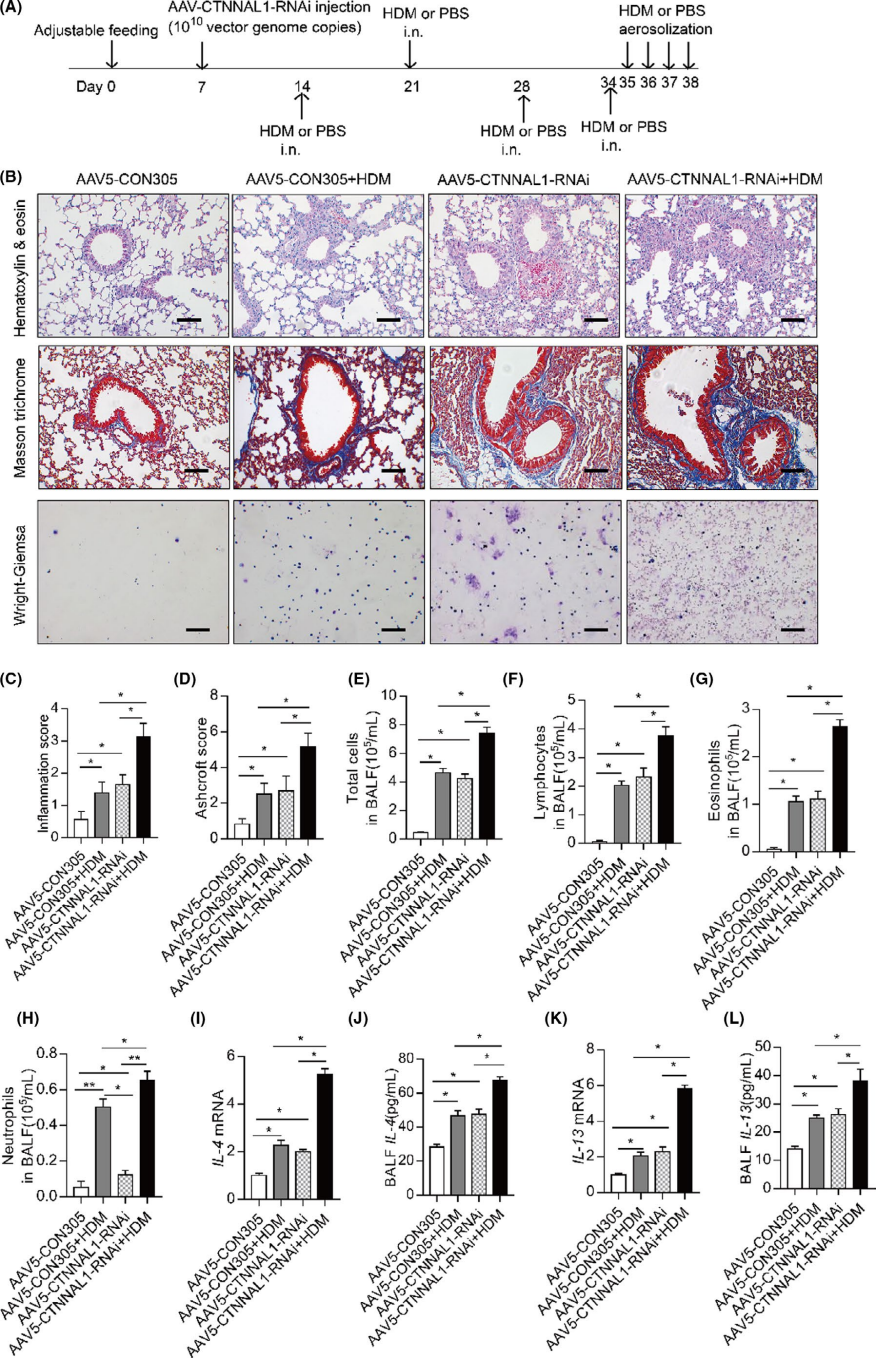
HDM stress promoted airway inflammation in CTNNAL1‐deficient mice. (A) A timeline of AAV injection, allergen sensitization and exposure in this study. (B) Lung histopathology in the AAV5‐CON305 or AAV5‐CTNNAL1 ‐RNAi mice exposed to HDM or PBS as a vehicle control. Lung sections were stained with HE (*n* = 8; ×200, Bars = 100 μm). Lung sections were stained with MASSON (*n* = 6; ×200, Bars = 100 μm). Giemsa‐stained BALF cells shown in the picture (*n* = 6; ×200, Bars = 100 μm). (C) Inflammation score was measured independently by three pathologists blinded to the experiment (*n* = 6). (D) Quantification of airway collagen area (*n* = 6). (E–H) The inflammatory cell profile in the BAL fluid was evaluated. Total cells, neutrophils, lymphocytes and eosinophils were enumerated (*n* = 6). (I,K) mRNA expression of IL‐4 and IL‐13 in the lungs was detected by RT‐qPCR (*n* = 5). (J,L) The IL‐4 and IL‐13 concentration in the BALF was detected using ELISA kits (*n* = 5). All data are presented as mean ± SD of three independent experiments. *p < 0.05 and **p < 0.01

We depicted our experimental protocol in Figure 2A. A larger number of inflammatory cells were observed in the perivascular, peribronchial and parenchymal tissues of the AAV5‐CTNNAL1‐RNAi+HDM mice than in other groups. In addition, HDM treatment or CTNNAL1 deficiency could increase the amount of inflammatory cells infiltrated into lung tissues respectively (Figure 2B,C).

There was an increased amount of collagen fibres in the AAV5‐CTNNAL1‐RNAi+HDM group compared with other groups. Larger amount of collagen was deposited in the AAV5‐CON305+HDM and AAV5‐CTNNAL1‐RNAi groups compared with AAV5‐CON305 group (Figure 2B,D). Furthermore, the inflammatory cells in the BAL fluid were evaluated (Figure 2B). The total number of cells in the BAL fluid of the AAV5‐CTNNAL1‐RNAi+HDM mice was higher than any other groups. The differential cell count analysis showed a larger number of lymphocytes, neutrophils and eosinophils in the BAL fluid of the AAV5‐CTNNAL1‐RNAi+HDM mice. The number of lymphocytes and eosinophils in the AAV5‐CON305+HDM mice and AAV5‐CTNNAL1‐RNAi mice were significantly higher than AAV5‐CON305 mice. In addition, the number of neutrophils in AAV5‐CON305+HDM group was larger than AAV5‐CON305 and AAV5‐CTNNAL1‐RNAi group (Figure 2E,H). The secretion of IL‐4 and IL‐13 significantly increased in the lungs of the HDM‐treated AAV5‐CTNNAL1‐RNAi group compared with other groups, and was significantly higher in CTNNAL1‐deficient group and HDM‐stressed AAV5‐CON305 group than in AAV5‐CON305 group (Figure 2I–L).

### 3.3 CTNNAL1 deficiency‐induced mucus hypersecretion in HDM‐treated mice

**FIGURE 3:**
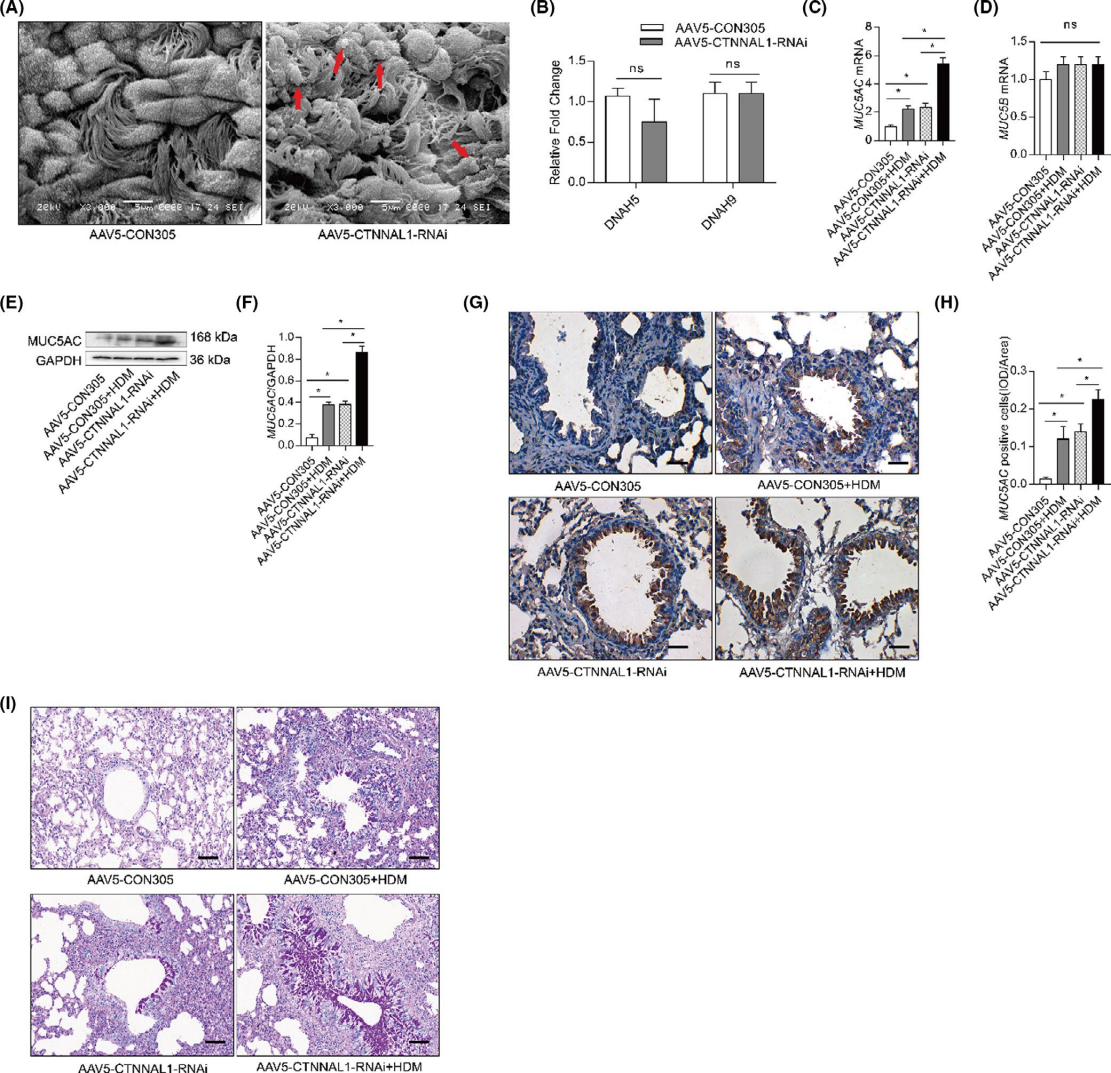
CTNNAL1 deficiency‐induced mucus hypersecretion in HDM‐treated mice. (A) Ciliated tracheae were analysed by scanning electron microscope in AAV5‐CON305 and AAV5‐CTNNAL1 ‐RNAi mice (*n* = 4). (B) mRNA expression of DNAH5 and DNAH9 in the lungs was detected by RT‐qPCR (*n* = 6). (C,D) mRNA expression of MUC5AC and MUC5B in the lungs was detected by RT‐qPCR (*n* = 5). (E,F) Protein expression of MUC5AC in the lungs was detected via Western blot (*n* = 5). (G, H) MUC5AC expression in the airway was detected by immunohistochemistry. (*n* = 5; ×400, Bars = 50 μm). (I) Mucus production of AB‐PAS‐stained airways was exposed to HDM. (*n* = 6; ×200, Bars = 100 μm). The experiments were performed three times, and the error bars represent means ± SD. *p < 0.05, ns:no significance

Furthermore, scanning electron microscope showed mucus production and the adhesion and lodging of cilia in the tracheae of AAV5‐CTNNAL1‐RNAi mouse group (Figure 3A). There was no difference in mRNA expression of DNAH5 and DNAH9, which encoded a component of the ciliary outer dynein arms (ODAs) and is essential for force generation during ciliary beating, between AAV5‐CON305 group and AAV5‐CTNNAL1‐RNAi group (Figure 3B). MUC5AC mRNA expression was elevated in the lungs of the HDM‐treated AAV5‐CTNNAL1‐RNAi mice (Figure 3C). However, the silencing of CTNNAL1 in addition to HDM exposure did not influence MUC5B expression (Figure 3D). The protein expression of MUC5AC was significantly increased in the HDM‐stressed CTNNAL1‐deficient mice. The expression of MUC5AC in the AAV5‐CTNNAL1‐RNAi and the AAV5‐CON305+HDM mice groups was higher than that of the AAV5‐CON305 group (Figure 3C,E,F), and consistent result was observed by immunohistochemistry (Figure 3G,H). Histological examination using PAS staining of lung tissues showed goblet cell hyperplasia and mucus overproduction in the airway mucosa of HDM‐challenged AAV5‐CON305 mice and CTNNAL1‐deficient mice compared with AAV5‐CON305 mice. There was a marked increase in mucus overproduction of HDM‐treated CTNNAL1‐deficient mice group compared with other groups (Figure 3I).

### 3.4 CTNNAL1 deficiency decreased the expression of RhoA and ROCK1 but increased the expression of ROCK2

**FIGURE 4:**
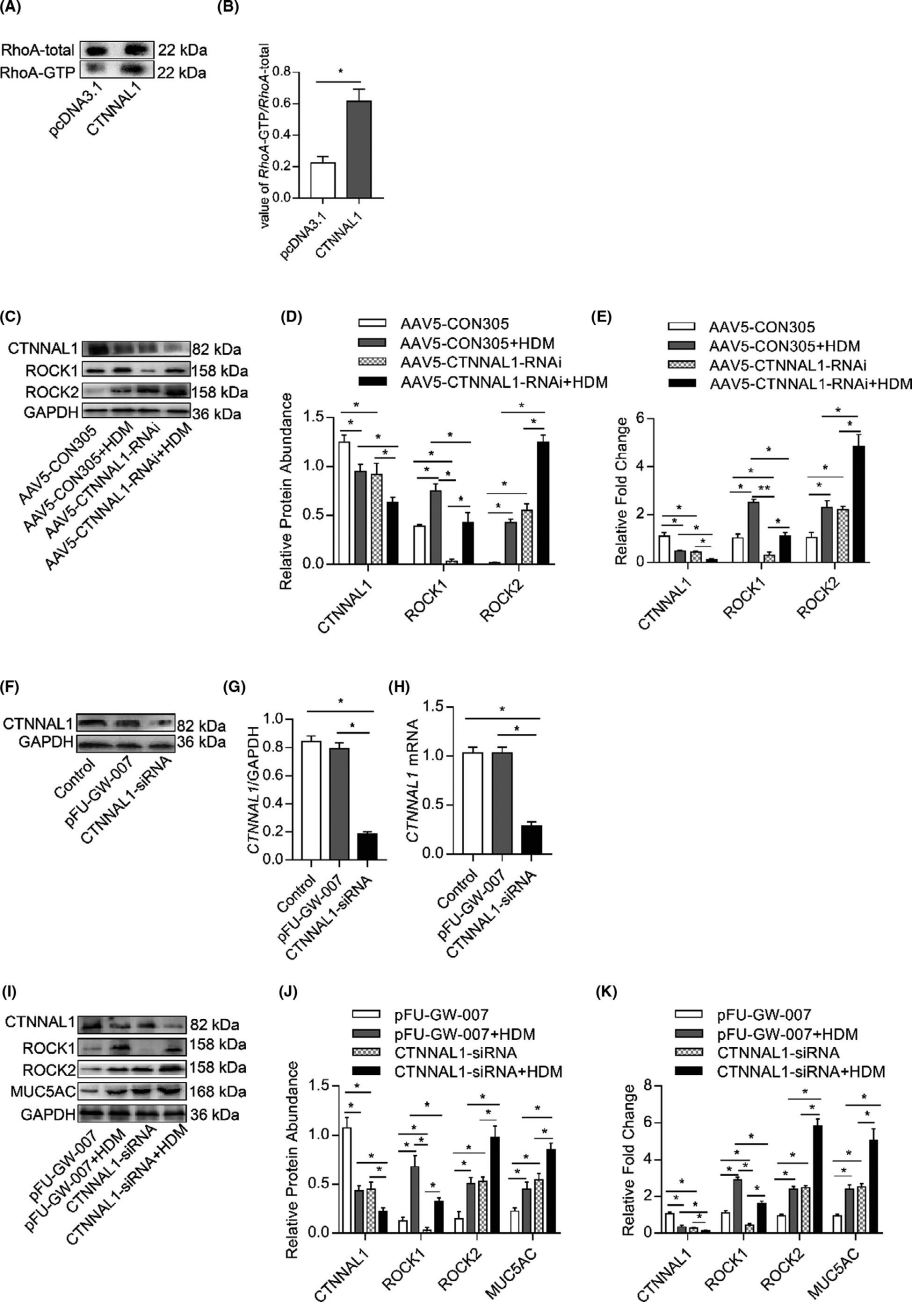
CTNNAL1 deficiency decreased the expression of RhoA and ROCK1 but increased the expression of ROCK2. (A,B) The expression of RhoA‐GTP and RhoA‐total was examined after CTNNAL1 was overexpressed by plasmid in 16HBE14o^−^ cells. RhoA activity was detected by a Rho activity assay kit (*n* = 5). (C,D) Lung tissues were obtained from HDM‐challenged AAV5‐CTNNAL1‐RNAi and AAV5‐CON305 groups after the last HDM challenge. Protein expression of CTNNAL1, ROCK1 and ROCK2 was detected by Western blotting (*n* = 8). (E) mRNA expression of CTNNAL1, ROCK1 and ROCK2 in the lungs was detected by RT‐qPCR (*n* = 6). (F,G) Protein expression of CTNNAL1 in 16HBE14o^−^ cells was assayed by Western blot (*n* = 8). (H) mRNA expression of CTNNAL1 in 16HBE14o^−^ cells was detected by RT‐qPCR (*n* = 6). (I,J) Protein expression of CTNNAL1, ROCK1, ROCK2 and MUC5AC in HDM‐treated 16HBE14o^−^ cells was detected by Western blot (*n* = 8). (K) mRNA expression of CTNNAL1, ROCK1, ROCK2 and MUC5AC in 16HBE14o^−^ cells was detected by RT‐qPCR. The experiments were performed three times, and the error bars represent means ± SD. *p < 0.05 and **p < 0.01

It is reported that Rho/ROCK inhibitor, fasudil, decreased the mucus hypersecretion in HDM‐treated mice.^28^ In addition, CTNNAL1 is part of the Rho signalling pathway. To reveal the mechanism by which CTNNAL1 deficiency results in mucus hypersecretion, we examined RhoA and ROCK expression. Firstly, we detected the expression of RhoA‐total and RhoA‐GTP after overexpressing CTNNAL1 by transfecting plasmid into 16HBE14o‐ cells. Our results demonstrated that the expression of RhoA‐GTP was enhanced by CTNNAL1 overexpression (Figure 4A,B). ROCK1 and ROCK2 are the downstream targets of RhoA. ROCK2 expression in the AAV5‐CTNNAL1‐RNAi+HDM group was higher than other groups. In addition, both CTNNAL1 deficiency and HDM stimulation could elevate the expression of ROCK2(Figure 4C–E). ROCK1 expression decreased when CTNNAL1 was silenced. However, HDM stimulation could elevate ROCK1 expression (Figure 4C–E). To further verify the different effects of CTNNAL1 had on the expression of ROCK1 and ROCK2 in vitro, we constructed CTNNAL1‐silenced HBE cells by small interfering RNA (siRNA) transfection (Figure 4F–H). ROCK1 expression decreased when CTNNAL1 was silenced and increased upon HDM stimulation (Figure 4I–K). The expression of ROCK2 significantly increased in HDM‐treated CTNNAL1‐siRNA group. Higher expression of MUC5AC was observed in the HDM‐treated CTNNAL1‐siRNA group compared with other groups. The expression of MUC5AC in CTNNAL1‐siRNA group and HDM‐treated control group was higher than control group (Figure 4I–K). The same results were observed in vivo.

To further verify that CTNNAL1 participates in the regulation of mucus overproduction, we overexpressed CTNNAL1 on HBE cells (Figure S2A–C). MUC5AC mRNA and protein expression were both decreased in CTNNAL1 group compared with pcDNA3.1 group. Further, MUC5AC expression was elevated after HDM stimulation. In addition, MUC5AC expression in CTNNAL1+HDM group was lower than pcDNA3.1+HDM group (Figure S2D–F). Moreover, ROCK2 expression significantly decreased in CTNNAL1 group compared with other groups. Furthermore, ROCK2 expression was elevated in CTNNAL1+HDM group compared with CTNNAL1 group (Figure 2D–F).

### 3.5 ROCK1 inhibition did not alter MUC5AC expression

**FIGURE 5:**
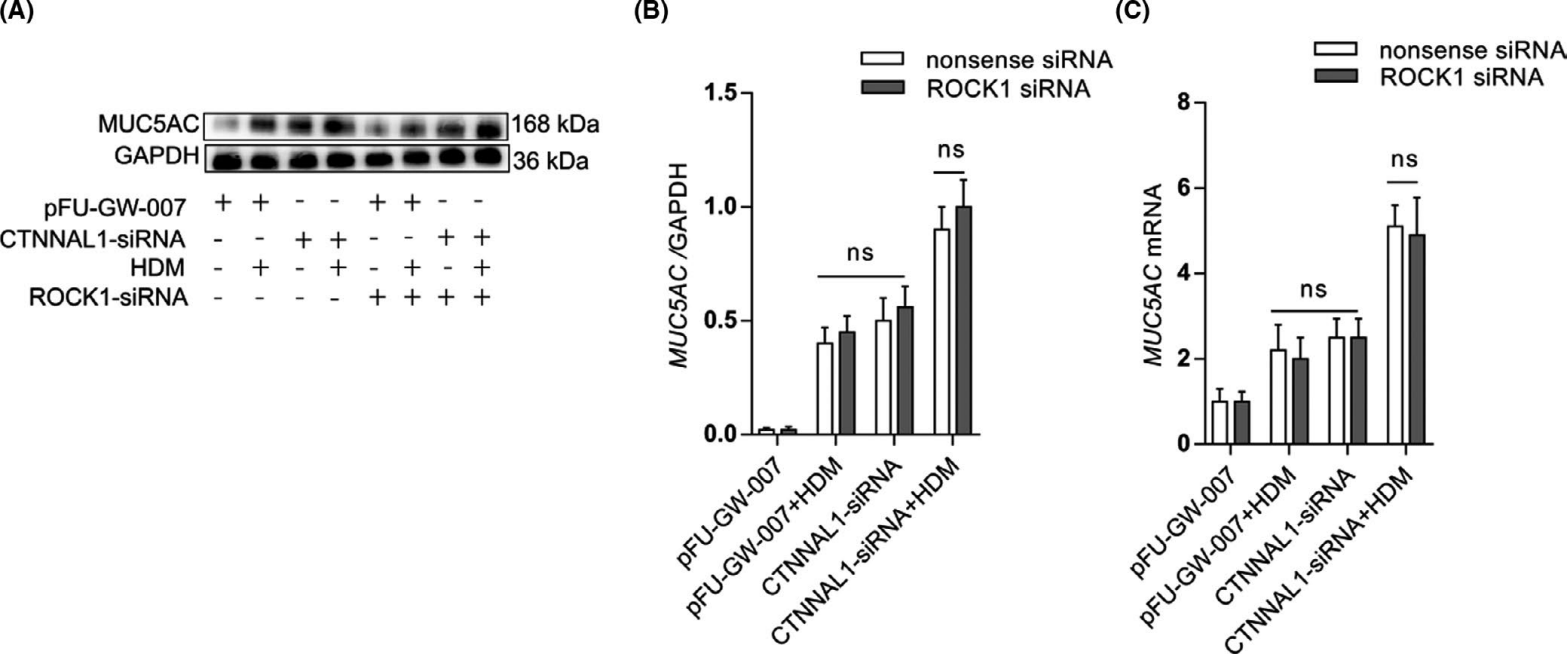
ROCK1 may not relate with mucus secretion. (A,B) We used ROCK1‐siRNA to inhibit ROCK1 in 16HBE14o^−^ cells. Protein expression of MUC5AC in 16HBE14o^−^ cells was detected via Western blot (*n* = 3). (C) mRNA expression of MUC5AC in 16HBE14o^−^ cells was detected via RT‐qPCR (*n* = 3). The experiments were performed three times, and the error bars represent means ± SD. ns, no significance

To investigate whether ROCK1 contributes to mucus production when CTNNAL1 was silenced, we inhibited ROCK1 by siRNA in airway epithelial cells. As shown in Figure 5, inhibition of ROCK1 did not influence MUC5AC expression in CTNNAL1‐deficient HBE cells (Figure 5A–C).

### 3.6 ROCK2 inhibition downregulated mucus secretion in response to CTNNAL1 deficiency

**FIGURE 6:**
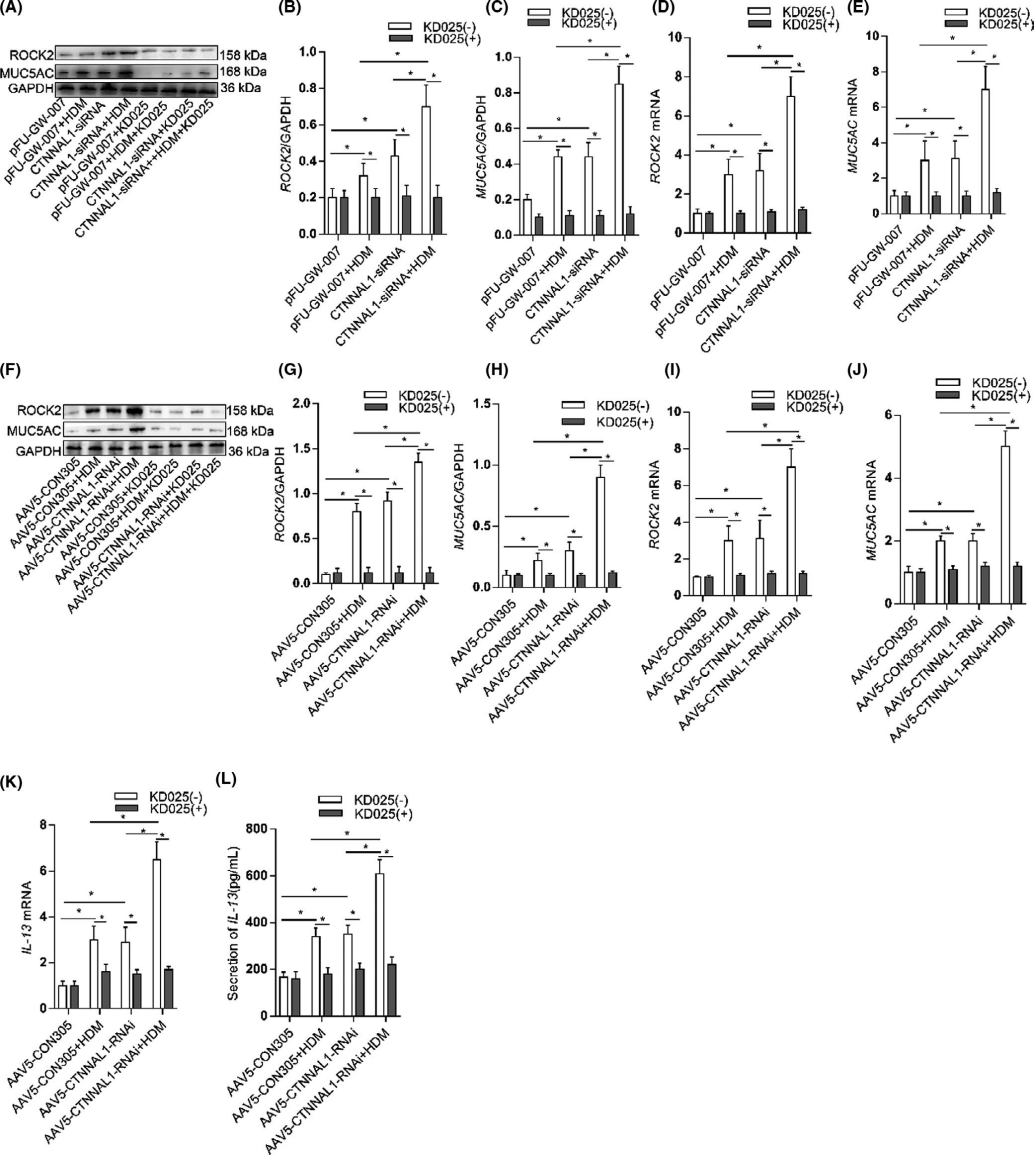
ROCK2 inhibition downregulated mucus secretion in response to CTNNAL1 deficiency. (A–C) We used KD025 to inhibit ROCK2 in 16HBE14o^−^ cells. Protein expression of ROCK2 and MUC5AC was detected by Western blot (*n* = 5). (D,E) mRNA expression of ROCK2 and MUC5AC in 16HBE14o^−^ cells was detected via RT‐qPCR (*n* = 5). (F–H) Protein expression of ROCK2 and MUC5AC in the tracheae was detected via Western blot (*n* = 6). (I–J) mRNA expression of ROCK2 and MUC5AC in the tracheae was detected by RT‐qPCR (*n* = 6). (K) mRNA expression of IL‐13 in tracheae was examined via RT‐qPCR (*n* = 4). (L) Concentration of IL‐13 was examined by ELISA (*n* = 4). The experiments were performed three times, and the error bars represent means ± SD. *p < 0.05 and **p < 0.01

To investigate whether ROCK2 contributes to mucus secretion when CTNNAL1 was silenced, we used the ROCK2 specific inhibitor‐KD025 to treat HBE cells. It effectively inhibited the mRNA and protein expression of ROCK2. In addition, KD025 decreased the mRNA and protein expression of MUC5AC in CTNNAL1‐silenced HBE cells with or without HDM treatment (Figure 6A–E).

Meanwhile, we took tracheae from CTNNAL1‐deficient mice and cultured them in serum‐free medium. KD025 reduced the MUC5AC expression in CTNNAL1‐deficient mice with or without HDM treatment (Figure 6F–J). IL‐13 level in culture supernatant was significantly increased in HDM‐stressed CTNNAL1‐deficient group. We also found that IL‐13 level was increased in HDM‐stressed control group and CTNNAL1‐deficient group compared with control group (Figure 6K,L). KD025 eliminated the effects that HDM and CTNNAL1 had on IL‐13 expression (Figure 6K,L).

### 3.7 CTNNAL1 regulates the expression of YAP

**FIGURE 7:**
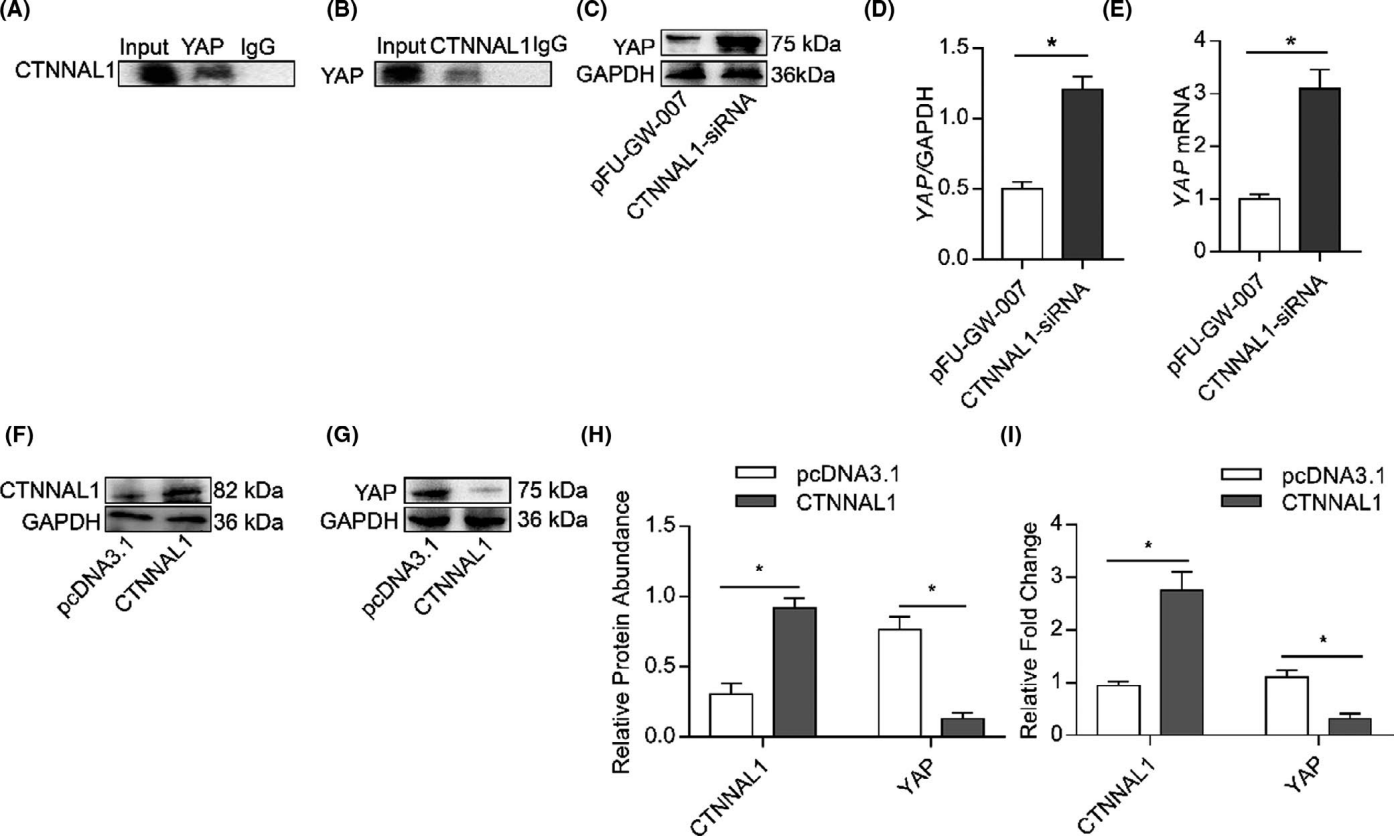
CTNNAL1 regulates YAP expression. (A,B) Interaction between CTNNAL1 and YAP was assessed by immunoprecipitation on airway epithelial cells (*n* = 5). (C,D) Protein expression of YAP in 16HBE14o^−^ cells was detected by Western blotting (*n* = 5). (E) mRNA expression of YAP in 16HBE14o^−^ cells was detected by RT‐qPCR (*n* = 6). (F–H) Protein expression of CTNNAL1 and YAP in 16HBE14o^−^ cells was detected via Western blotting (*n* = 5). (I) mRNA expression of CTNNAL1 and YAP in 16HBE14o^−^ cells was detected via RT‐qPCR (*n* = 6). The experiments were performed three times, and the error bars represent means ± SD. *p < 0.05 vs pFU‐GW‐007 group. *p < 0.05 and **p < 0.01

Our results demonstrated that ROCK2 inhibition downregulated mucus secretion when CTNNAL1 was silenced. However, ROCK1 inhibition did not alter MUC5AC expression. It is reported that YAP could interact with the promoter region of ROCK2.^31^ Thus, we sought to determine the possible involvement of the YAP‐mediated signalling pathways in CTNNAL1‐regulated ROCK2 production. The interaction between CTNNAL1 and YAP within the airway epithelial cells was detected via immunoprecipitation (Figure 7A,B). When CTNNAL1 was silenced, YAP expression was significantly increased (Figure 7C–E). Meanwhile, Western blot and RT‐qPCR analysis showed that CTNNAL1 overexpression‐induced YAP expression levels were decreased (Figure 7F–I).

### 3.8 CTNNAL1 deficiency increased the expression of ROCK2 via the YAP pathway

**FIGURE 8:**
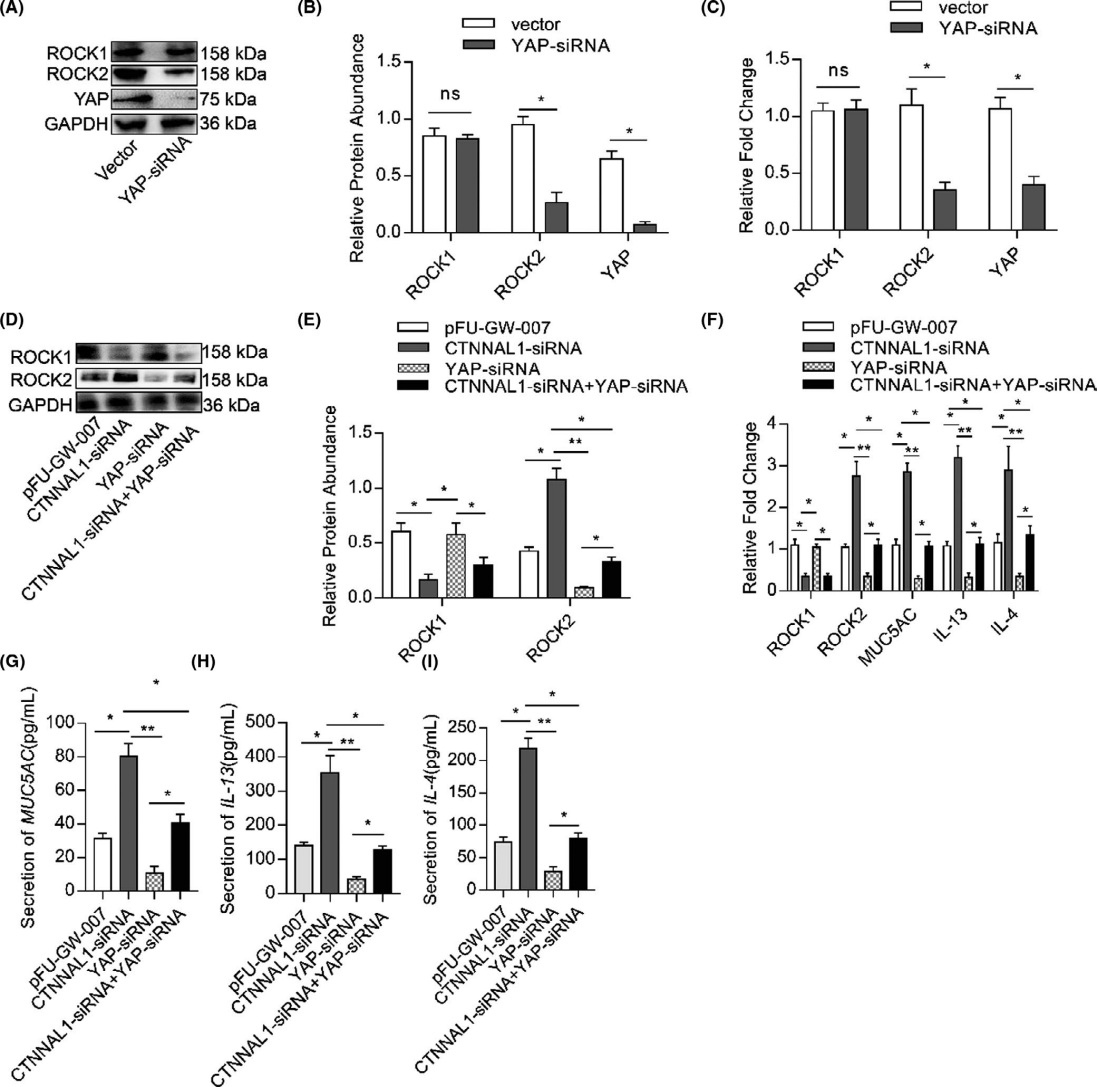
CTNNAL1 deficiency increases the expression of ROCK2 through the YAP pathway. (A–B) Protein expression of ROCK1, ROCK2 and YAP in 16HBE14o^−^ cells was detected via Western blotting (*n* = 6). (C) mRNA expression of ROCK1, ROCK2 and YAP in 16HBE14o^−^ cells was detected by RT‐qPCR (*n* = 6). (D,E) Protein expression of ROCK1 and ROCK2 was detected by Western blotting (*n* = 6). (F) mRNA expression of ROCK1, ROCK2, MUC5AC, IL‐13 and IL‐4 was detected by RT‐qPCR (*n* = 6). (G–I) The MUC5AC, IL‐13 and IL‐4 concentration in the cellular supernatant was assayed by ELISA (*n* = 5). The experiments were performed three times, and the error bars represent means ± SD. *p < 0.05, **p < 0.01, ns:no significance

To further verify the involvement of the YAP signalling pathway in CTNNAL1 deficiency‐induced mucus overproduction, we silenced YAP by siRNA transfection. It decreased the transcription level of ROCK2 but did not affect ROCK1 expression level (Figure 8A–C). Inhibition of YAP did not alter ROCK1 expression in CTNNAL1‐deficient HBE cells (Figure 8D–F). ROCK2 expression was significantly increased in the CTNNAL1‐siRNA groups compared with other groups. Meanwhile, inhibition of YAP led to a reduction of ROCK2 expression. When silencing both CTNNAL1 and YAP, the expression of ROCK2 was higher than YAP‐siRNA group but lower than CTNNAL1‐siRNA group (Figure 8D–F). We detected the ROCK2‐regulated molecules expression including MUC5AC, IL‐13and IL‐4 in the cell culture medium of each group. Their expression was increased in the CTNNAL‐siRNA group. Moreover, after YAP was silenced, their expression decreased, while manifested intermediate expression level when silencing both YAP and CTNNAL1(Figure 8G–I).

## 4 DISCUSSION

In this study, we found that CTNNAL1 deficiency stimulated airway inflammation and increased inflammatory cells, including eosinophils, neutrophils and lymphocytes in the BALF. Especially, mucus production significantly increased in CTNNAL1‐deficient mice.

Mucus is an essential component of both the physiological and pathological processes in the airway.^38^, ^39^ Airway mucus plugging can affect asthma progression and aggravation, leading to death.^40^, ^41^, ^42^ Under asthmatic conditions, excessive production of MUC5AC contributes to mucous plugs and airflow obstruction.^43^, ^44^ In this study, we detected MUC5AC expression, which was significantly elevated in the lungs of HDM‐treated AAV5‐CTNNAL1‐RNAi mice. Meanwhile, the MUC5AC expression was increased in the lungs of HDM‐induced AAV5‐CON305 mice and CTNNAL1‐deficient mice compared with AAV5‐CON305 mice. Then, we found that CTNNAL1 deficiency elevated the levels of IL‐13 and IL‐4 in the mice exposed to HDM. Moreover, the secretion of IL‐4 and IL‐13 in CTNNAL1‐deficient mice and HDM‐exposed control mice was higher than that in control mice. It is reported that IL‐13 and IL‐4, cytokines released from Th2, are major drivers of asthma and appear to play a prominent role in MUC5AC expression and mucus production.^45^, ^46^ Th2 cytokines induce goblet cell hyperplasia, resulting in mucus overproduction and airway inflammation in allergic asthma.

CTNNAL1 was identified as a part of the Rho signalling pathway, serving as a scaffold protein for Lbc.^24^ We have found that CTNNAL1 enhances RhoA activity. ROCK is a downstream effector of RhoA. There are two different ROCK forms, ROCK1 and ROCK2, which are structurally similar, particularly in their kinase domains where they are 90% homologous. ROCK1 and ROCK2 have different functions in various types of cells and tissues.^47^, ^48^ In our study, when CTNNAL1 was silenced, the expression of ROCK1 decreased while ROCK2 increased both in vitro and in vivo. We found that CTNNAL1 deficiency significantly increased ROCK2 expression after HDM stimulation.

Moreover, our results demonstrated that inhibition of ROCK1 did not influence MUC5AC expression in vitro. Inhibition of ROCK2 decreased MUC5AC expression in CTNNAL1‐defective mice and CTNNAL1‐defective 16HBE14o‐ cells. Moreover, administration of ROCK2 inhibitor‐KD025 significantly blocked the MUC5AC expression after HDM exposure both in tracheae, which was cultured in medium and in HBE cells. At the same time, KD025 decreased the secretion of IL‐13 and IL‐4 in CTNNAL1‐deficient mice. More importantly, KD025 treatment blocked IL‐4 and IL‐13 secretion in CTNNAL1‐silenced mice after HDM exposure. These results demonstrated that inhibition of ROCK2 led to the reduction of mucus and cytokines secretion when CTNNAL1 was silenced. In addition, it has been reported that an increase in ROCK2 leads to mucus hypersecretion,^49^, ^50^ which confirmed our results.

**FIGURE 9:**
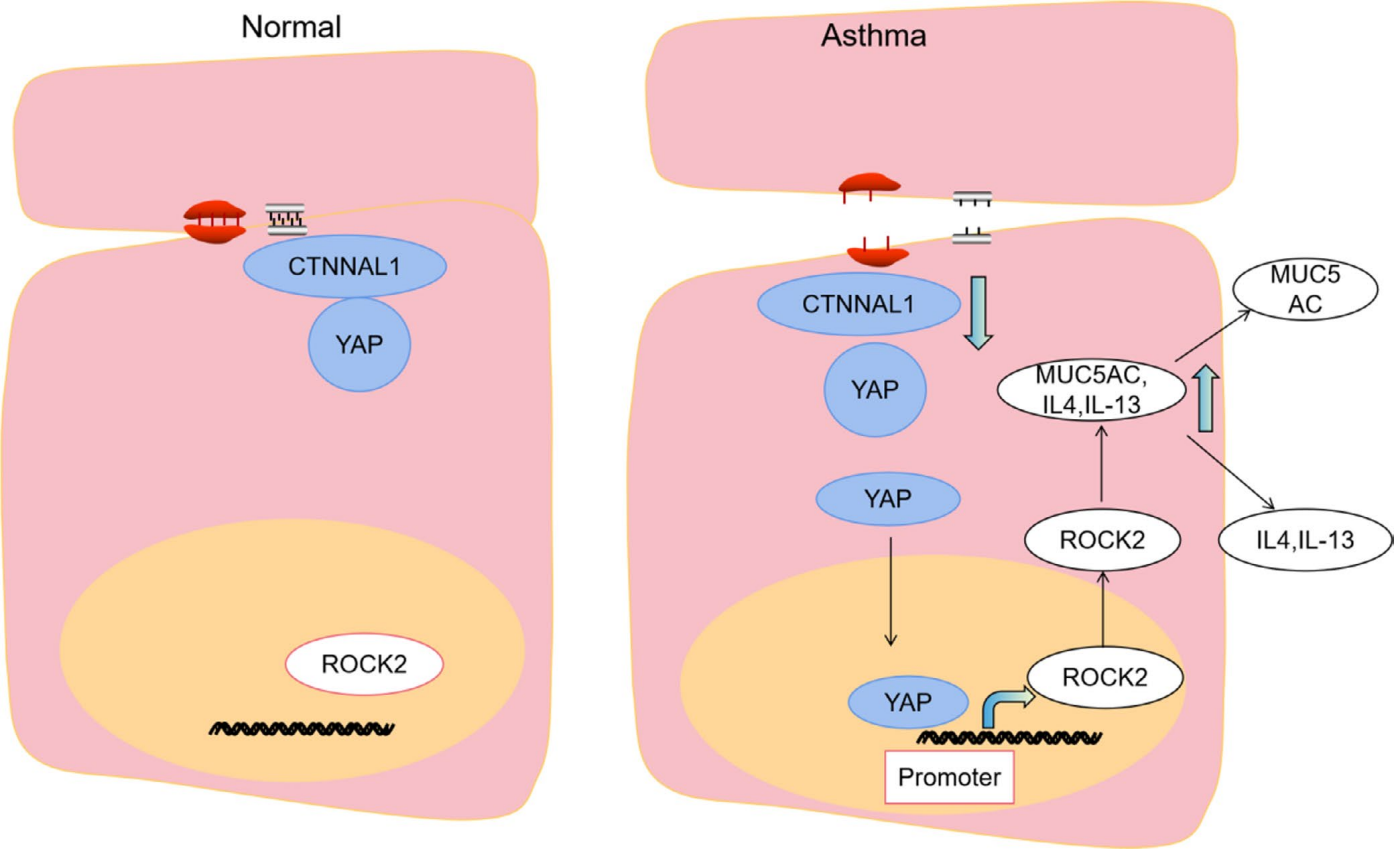
Schematic model of CTNNAL1 mediating mucus hypersecretion through YAP‐ROCK2 signalling pathway. In asthmatic model, CTNNAL1 deficiency stimulated YAP expression, which translocated into nucleus and bound with ROCK2 promoter. ROCK2 led to the expression of MUC5AC, IL‐4 and IL‐13 increased, which secreted from airway epithelial cells into airway lumen

Although ROCK1 and ROCK2 share more than 90% homology in their kinase domains, they had different expression when CTNNAL1 silenced. ROCK1 and ROCK2 might be regulated by different transcription factors when CTNNAL1 silenced. Wataru Sugimoto et al. reported that YAP interacted with the ROCK2 promoter region. α‐catenin is a key protein of adherent junctions (AJs) with mechanosensory properties. Karin Schlegelmilch et al. reported that α‐catenin controls Yap1 activity and phosphorylation by modulating its interaction with 14–3–3 protein and the PP2A phosphatase.^51^ Mark et al. confirmed that YAP interacts with α‐catenin.^52^ CTNNAL1 is highly homologous to alpha‐catenin, and we confirmed the interaction between CTNNAL1 and YAP by Co‐IP. Results showed that CTNNAL1 deficiency could activate the expression of YAP. Correspondingly, when CTNNAL1 was overexpressed, the expression of YAP decreased. In order to further confirm the participation of YAP in mucus secretion induced by CTNNAL1 deficiency, we blocked YAP expression in CTNNAL1‐silenced airway epithelial cells. Our results showed that YAP inhibition could decrease MUC5AC, IL‐4 and IL‐13 expression in CTNNAL1‐deficient airway epithelial cells. These results demonstrated that CTNNAL1 deficiency‐induced mucus overproduction through YAP signalling pathway. Based on foresaid results, we sought to build the connection between YAP and ROCK2. ROCK2 expression was elevated when CTNNAL1 was silenced while decreased after YAP silenced. When YAP and CTNNAL1 were silenced together, ROCK2 expression returned to normal level. It indicated us that CTNNAL1 deficiency promoted mucus overproduction through YAP‐ROCK2 signalling pathway (Figure 9).

## 5 CONCLUSIONS

In summary, we demonstrated that CTNNAL1 deficiency enhanced lung inflammation and mucus hypersecretion through the activation of the YAP‐ROCK2 signalling pathway in both lung tissue and in HBE cells, indicating CTNNAL1 could be a therapeutic target of mucus secretion in the future.

## CONFLICT OF INTEREST

The authors declare no conflict of interest.

## AUTHOR CONTRIBUTIONS

**Di Wu:** Methodology (equal); Visualization (equal); Writing – original draft (lead). **Wang Jiang:** Investigation (equal). **Caixia Liu:** Formal analysis (equal). **Lexin Liu:** Software (equal). **Furong Li:** Methodology (equal). **Xiaodi Ma:** Software (equal). **Lang Pan:** Supervision (equal). **Chi Liu:** Funding acquisition (equal). **Xiangping Qu:** Supervision (equal). **Huijun Liu:** Visualization (equal). **Xiaoqun Qin:** Conceptualization (equal); Funding acquisition (equal). **Yang Xiang:** Conceptualization (equal); Funding acquisition (equal); Resources (equal); Validation (equal).

## Supporting information

Fig S1‐S2

## Data Availability

The data presented in this study are available on request from the corresponding author.
