# Supplementary material for: CTNNAL1 participates in the regulation of mucus overproduction in HDM‐induced asthma mouse model through the YAP‐ROCK2 pathway
Source: J Cell Mol Med. 2022 Jan 28;26(5):1656–71. doi: 10.1111/jcmm.17206 (PMC8899158; doi:10.1111/jcmm.17206)
Supplement: Supplementary file 1 — Fig S1‐S2 [file JCMM-26-1656-s001.docx]

Supplementary figure 1

**
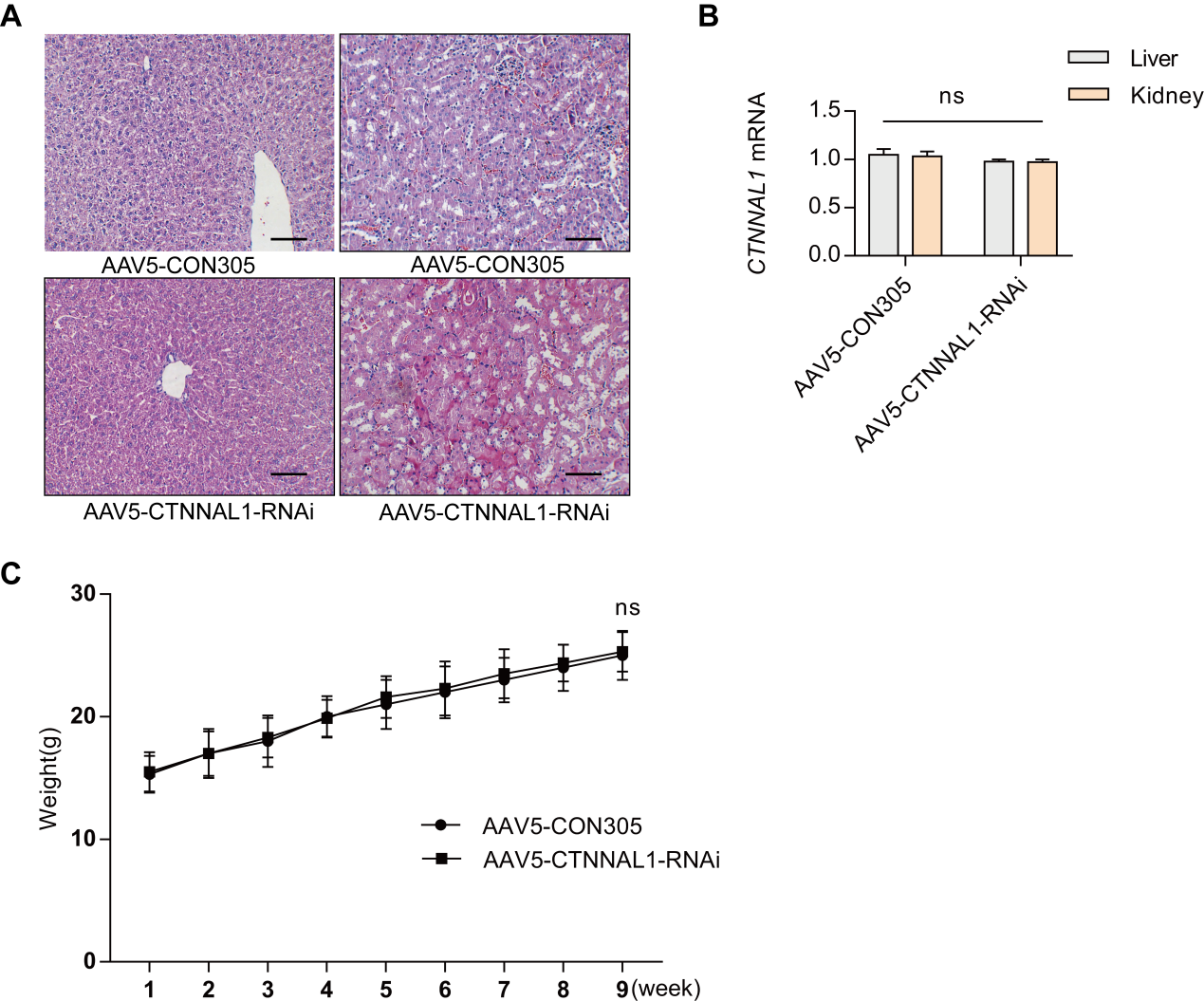
**

**Supplementary Figure 1** (A)Liver and kidney histopathology in AAV5-CON305 or AAV5-CTNNAL1-RNAi mice(n=6; x200, Bars=100 *μ*m). (B) CTNNAL1 mRNA expression from liver and kidney homogenate was detected via RT-qPCR(n=8). (C) Mice weight after 8 weeks of AAV-CTNNAL1-RNAi injections(n=8).The experiments were performed three times and the error bars represent means ± SD. ns:no significance.

Supplementary figure 2


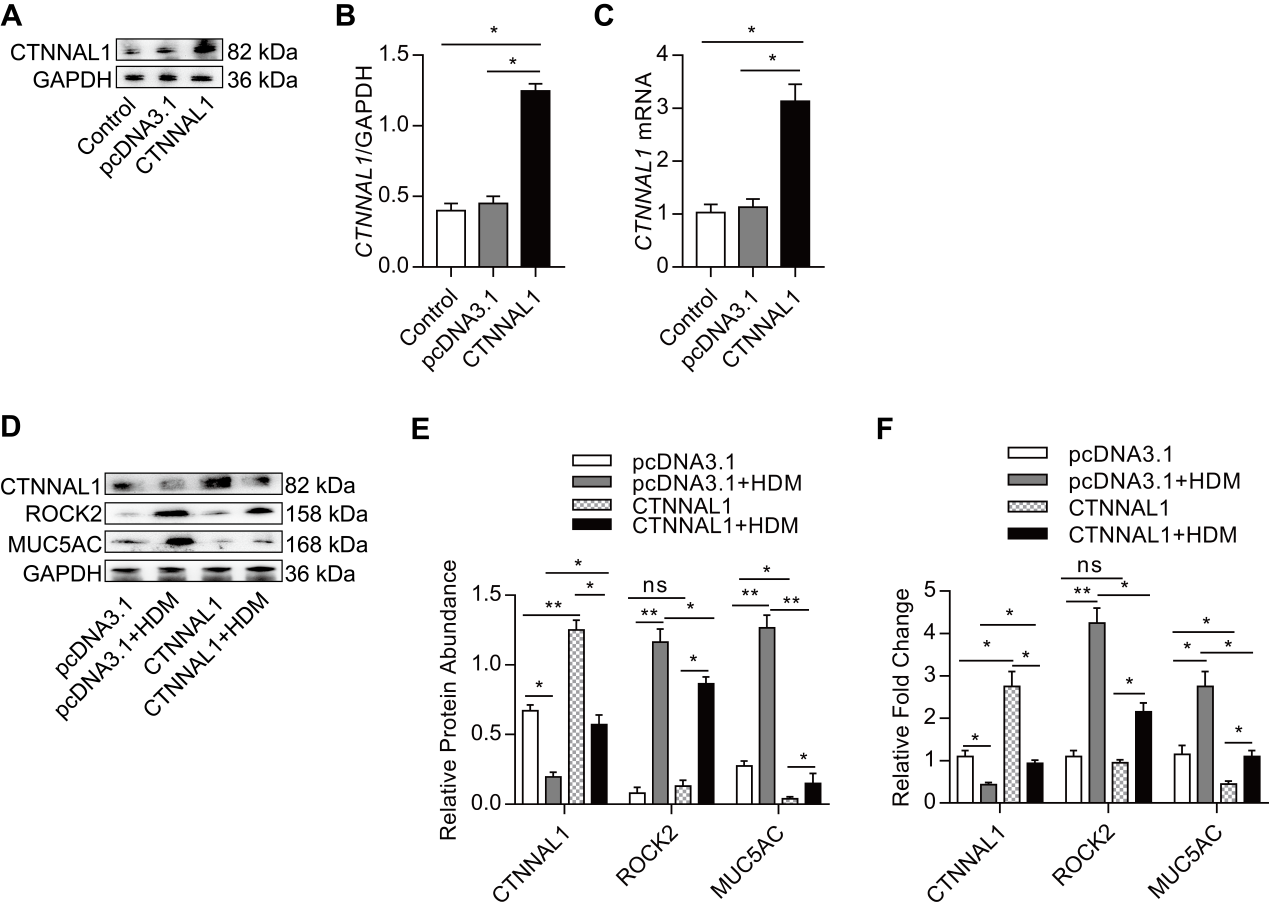


**Supplementary Figure 2** (A,B)CTNNAL1 protein expression in the 16HBE14o^-^ cells was detected by Western blot. Quantification of western blotting normalized to the level of glyceraldehyde 3-phosphate dehydrogenase (GAPDH)(n=5).(C)CTNNAL1 mRNA expression in the 16HBE14o^-^ cells was detected via RT-qPCR(n=5).(D,E)Protein expression of CTNNAL1,MUC5AC and ROCK2 in the 16HBE14o^-^ cells was detected by Western blot(n=6).(F)mRNA expression of CTNNAL1,MUC5AC and ROCK2 in the 16HBE14o^-^ cells was detected via RT-qPCR(n=6).The error bars represent means ± SD. **P*<0.05 and ^**^*P*<0.01,ns:no significance.
